# Synaptic Changes in Mice Lacking Alpha- and Gamma-Synucleins

**DOI:** 10.3390/biomedicines13122866

**Published:** 2025-11-25

**Authors:** Anastasia M. Krayushkina, Olga Morozova, Anastasia Khizeva, Tamara A. Ivanova, Natalia Ninkina, Kirill Chaprov

**Affiliations:** 1 Institute of Physiologically Active Compounds at Federal Research Center of Problems of Chemical Physics and Medicinal Chemistry, Russian Academy of Sciences, 142432 Chernogolovka, Russia; 2 School of Biosciences, Cardiff University, Sir Martin Evans Building, Museum Avenue, Cardiff CF10 3AX, UK

**Keywords:** Parkinson’s disease, alpha-synuclein, beta-synuclein, gamma-synuclein, striatum, conditional knock-out, mouse model

## Abstract

**Background:** Alpha-synuclein is a key protein involved in the pathogenesis of Parkinson disease (PD). Its intermediate aggregated forms disturb the normal function of dopaminergic (DA) neurons. Furthermore, the loss of intraneuronal connections may precede nerve cell death in PD. Disturbance of presynaptic functions of alpha-synuclein and its family members, beta- and gamma-synuclein, can apparently be the first step in nigrostriatal system dysfunction. Based on their structure homology and subcellular localization, the three synuclein proteins could have overlapping functions. This also indicates necessitates to study each protein in isolation. **Methods:** We have established a unique mouse line with conditional knockout (KO) of alpha-synuclein inactivation on the background of gamma-synuclein KO. **Results:** During the early phase of alpha-synuclein loss of function, mice demonstrate reduced explorer activity, decreased gene expression of *Mao-B* in the midbrain, and transiently increased levels of beta-synuclein protein in the striatum after alpha-synuclein inactivation, as results, metabolism of dopamine stays unscathed. These changes can be caused by specific regulation of Mao-B by alpha-synuclein or can be a physiological reaction aimed at restoring alpha-synuclein levels. No significant changes in gene expression patterns of dopamine-related enzymes in the midbrain or protein levels in the striatum and midbrain were observed. **Conclusions:** Our data suggest that sudden alpha-synuclein depletion leads to an increase in beta-synuclein levels, likely as functional replacement. This result supports that beta-synuclein can compensate the loss of alpha-synuclein. In general, this process may characterize synapse reconstruction in early alpha-synuclein dysfunction with gamma-synuclein absence and form the basis for replacement therapeutic strategies in PD.

## 1. Introduction

Parkinson’s disease (PD), a common neurodegenerative disease characterized by a progressive, irreversible course, results in severe disability and increases the global healthcare burden year after year [1]. Pathognomonic for PD is the formation of Lewy bodies, protein aggregates containing alpha-synuclein, in the dopaminergic (DA) neurons of the substantia nigra (SN). Variations mutations in the *Snca*, the alpha-synuclein coding gene, are the main risk factor for the development of the disease [2]. The toxic effect of aggregated forms of alpha-synuclein causes the selective death of DA neurons of the SN, resulting in dopamine deficiency [3]. The lack of neurotransmitter affects the impairment of movement control and leads to bradykinesia, rigidity, resting tremor, and postural instability—the main signs of PD [4]. However, the prodromal period of PD can begin up to 15–20 years before the first motor symptoms and is currently considered a stage of PD [5]. A series of preclinical studies has established that the pathologic process begins with synaptic dysfunction [6,7,8] and that loss of intraneuronal connections may precede nerve cell death. Therefore, the focus of modern PD research has turned to all aspects of the disruption of presynaptic dopamine trafficking [9,10,11] and the role of alpha-synuclein in it, as well as two other proteins of the family—beta- and gamma-synuclein—which have been shown to be involved in neurotransmission in addition to alpha-synuclein [12].

Information transfer between neurons of the dopaminergic system is mediated by dopamine, the main neurotransmitter of the vertebrate CNS involved in learning, reward, motor control, and emotion [13]. In the brain, dopamine is synthesized in DA neurons of the SN and VTA, which have different projections and play an important role in the functioning of the brain’s dopaminergic system. Thus, the prefrontal (PFC), cingulate, and perirhinal cortices act as the main projections of the DA neurons of the ventral tegmental area (VTA) and form a mesocortical pathway [14]. Dopaminergic effects on the PFC are necessary for higher cognitive functions such as working memory, attention, planning, and decision making [15]. In addition to the cortex, dopamine is released from VTA neurons into the nucleus accumbens (ventral striatum) of the limbic system via the amygdala and hippocampus, forming the mesolimbic pathway responsible for reward and emotion. Neurites of the SN pars compacta (SNpc) DA neurons are directed to the dorsal striatum (caudate nucleus/scores), where the synapses of the corresponding neurons are located [16,17]. This pathway plays a leading role in learning new motor skills and controlling motor functions. Parkinson’s motor dysfunction is caused by gradual degeneration of this specific group of DA neurons [18].

DA synthesis occurs in the cytosol of DA neurons: tyrosine is hydroxylated by tyrosine hydroxylase (TH) to L-dihydroxyphenylalanine (L-DOPA) and then decarboxylated by aromatic amino acid decarboxylase (AADC) to dopamine [19]. Elevated levels of alpha-synuclein have been shown to reduce dopamine synthesis by decreasing TH synthesis and activity through direct interaction in DA neurons [20]. The activity of TH isolated from the adrenal gland was also inhibited by recombinant human alpha-synuclein in a dose-dependent manner [21]. After synthesis dopamine is immediately packaged into vesicles by vesicular monoamine transporter 2 (VMAT-2). Beta-synuclein interacts with VMAT-2 and promotes the optimization of dopamine uptake by synaptic vesicles [22]. Electrophysiological stimulation of neurons causes exocytosis of synaptic vesicles and resulting in the release of DA into the synaptic cleft [23]. Once DA has interacted with receptors, it is released from its bond with them. The neurotransmitter can then undergo both reuptake by DA neurons [24] and uptake by and degradation in glial cells. Dopamine reuptake occurs with the participation of the dopamine transporter protein (DAT) [25,26]. Alpha-, beta-, and gamma-synucleins modulate the expression and function of monoamine transporters, thus regulating monoamine reuptake [27]. The degradation of dopamine occurs by two parallel pathways [28]; see schemes in paper [29] and figures 2 and 4, in publications [30,31], respectively. In the first, dopamine is metabolized to 3,4-dihydroxyphenylacetaldehyde (DOPAL) by monoamine oxidase-A (Mao-A) or monoamine oxidase-B (Mao-B). DOPAL is in turn metabolized by aldehyde dehydrogenase (ALDH) to 3,4-dihydroxyphenylacetic acid (DOPAC) and then by catechol-o-methyltransferase (COMT) to homovanilic acid (HVA) [32]—one of the main degradation products of dopamine, which is excreted in the urine.

Dopamine metabolism via the second pathway involves the conversion of dopamine by the COMT to 3-methoxytyramine (3-MT), the major extracellular DA metabolite, which is then metabolized by Mao to HVA [33]. It is traditionally thought that both Mao-A and Mao-B are equivalently involved in DA degradation, conflicting data exists regarding their activity and respective roles in the DA metabolic pathway [31,34,35]. Nevertheless, to be deaminated by Mao-B, it is necessary for DA to first to enter the astrocytes, where this enzyme is mainly localized [36], whereas Mao-A is primarily localized to catecholaminergic neurons and is believed to be responsible for the metabolism of dopamine in DA neurons [37]. In vitro studies have shown that alpha-synuclein binds selectively to Mao-B but not to Mao-A, stimulating its enzymatic activity [38].

Thus, several enzymes and transporter proteins control the concentration of extracellular dopamine. Nevertheless, dopamine neurotransmission may be affected at the stage of neurotransmitter release from vesicles. Disruption of the synaptic vesicle fusion mechanism is often observed even before the debut of Parkinson’s disease and other synucleinopathies. The fusion of a vesicle to the presynaptic membrane includes a series of sequential steps: targeting, tethering, priming, and triggering the fusion event [39,40]. Although many proteins are involved in this process, the main ones are the so-called SNARE proteins—vesicle-associated membrane protein (VAMP), synaptobrevin, SNAP-25 (synaptosomal-associated protein, 25-kD), and syntaxin. They are subdivided into tSNAREs and vSNAREs depending on their location on the target plasma membrane or vesicle membrane, respectively [41]. By interacting with each other, these proteins form a highly stable complex consisting of four chains: one α-helix each from syntaxin1A and VAMP2 and two from SNAP-25 [42]. As a result of the SNARE complex assembly, the two membranes converge and become more curved [43]. Once the convergence has reached a maximum level, the membranes are hemifused, followed by the opening and expansion of the fusion pore [44]. In 2010, Sudhof and colleagues found that alpha-synuclein promotes the assembly of the SNARE complex by binding to vesicular VAMP2 [45,46]. Furthermore, alpha-synuclein stabilizes the complex, thereby regulating neurotransmission [47]. In vitro studies support these findings, indicating that alpha-synuclein is responsible for SNARE-dependent vesicle binding [48,49] and for the expansion of the fusion pore through its membrane-binding activity [40,50].

Although the role of alpha-synuclein in dopamine neurotransmission is well established [20,21,51], the roles of beta- and gamma-synuclein in this process are still less understood. Alpha- and beta-synuclein are characterized by the highest sequence similarity within the synuclein family and have a predominantly identical pattern of expression in the brain [12]. In an experiment in mouse models of constitutive knockout (KO), increased levels of beta-synuclein in the midbrain were shown in animals with both single and double KO of alpha- and gamma-synuclein genes compared to wild-type mice [52]. In addition, the combined absence of alpha- and beta-synuclein, but not just one of these proteins, worsened the pathological state in KO mice of the CSPα (cysteine string protein) gene. The protein encoded by this gene exhibits a chaperone function similar to alpha-synuclein [53]. The totality of these data suggests the existence of some compensatory mechanism between alpha- and beta-synucleins, which may become a new therapeutic strategy for PD.

**Figure 1:**
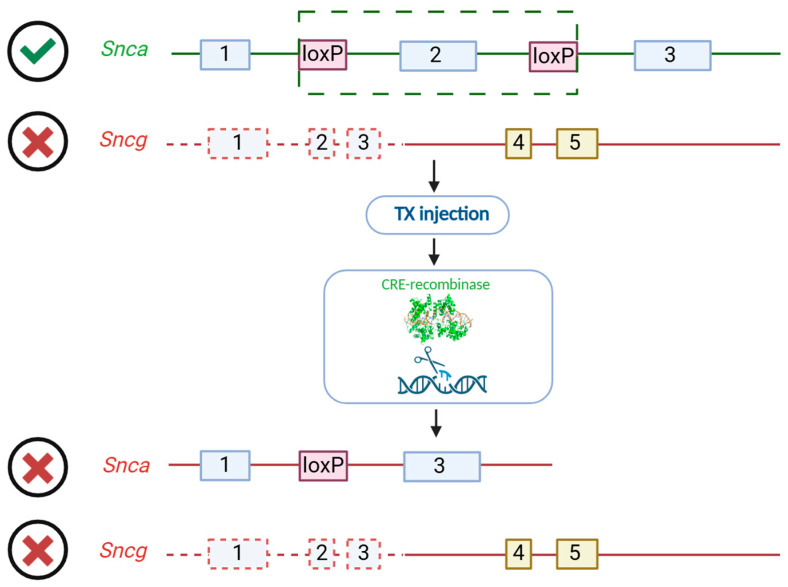
Scheme of conditional inactivation of *Snca* against the *Scng* KO background. Green box identifies CRE-recombination locus of *Snca* gene.

Nevertheless, the functions of gamma-synuclein are currently poorly understood, and its high degree of homology with alpha-synuclein raises the possibility of the compensatory potential on the part of gamma-synuclein. Therefore, in order to exclusively study the physiological functions of beta-synuclein, we have created a unique mouse line with the ability to conditionally inactivate alpha-synuclein against the background of gamma-synuclein KO (Figure 1). Previous studies have shown that 3 months are necessary and sufficient for completely alpha-synuclein clearing from tissues after tamoxifen injections [54]. In the mouse genome, the second exon of the alpha-synuclein coding gene is flanked by two loxP sites, and the transgenic cassette NSE-Cre-ER-T2 encodes a Cre recombinase under the neuro-specific enolase (NSE) promoter conjugated to the estrogen receptor T2 (ER-T2). Tamoxifen injections trigger the recombination process exclusively in neuronal tissues [55]. Due to the important role of alpha-synuclein in neurotransmission, its dysfunction can lead to disturbance in the associated proteins. Alternatively, no changes may occur because of the compensatory potential activation of beta-synuclein under conditions of other synuclein deficiency. Additionally, compensatory mechanisms are most effectively activated in young animals, so the age of mice at the beginning of our experiment was 6 months. Therefore, here we investigated whether there are compensatory changes in synapse during the critical 3-month period after *Snca* inactivation and whether the level of beta-synuclein, the only functional synuclein in model mice, is subject to fluctuations.

## 2. Materials and Methods

### 2.1. Experimental Animals

For creating the experimental cohort core, L3-*Snca ^flox/flox^*/*Sncb^+^*/*Sncg^−^*—(B6-*Snca*^tm1.1Vlb^ *Sncb*^tm1.1Sud^*Sncg*^tm1Vlb^/J) and subsidiary L7-*Snca ^delta_flox/delta_flox^*/*Sncb^+^*/*Sncg^−^*/tg-NSE-Cre-ER-T2Homo (B6-*Snca*^tm1.2Vlb^ *Sncb*^tm1.1Sud^ *Sncg*^tm1Vlb^/tg CRE-NSE-ER-T2/J) lines were crossed. To generate these lines, we previously used models obtained from JAX Stock 1.1025636 for B6-*Snca*^tm1.1Vlb^/J or JAX Stock 028559 for B6-*Snca*^tm1.2Vlb^/J, JAX Stock 008133 B6-*Sncb*^tm1.1Sud^/J, and JAX Stock 008843 B6- *Sncg*^tm1Vlb^/J from The Jackson Laboratory (Bar Harbor, ME, USA) and our Bioresource Collection of IPAC RAS and Centre for Collective Use (FFSG-2024-0020). F1 littermates had a mixed genotype *Snca ^flox/delta flox^*/*Sncb^+^*/*Sncg^−^*/tg-NSE-Cre-ER-T2_Hemi. Thereby, animals’ genome contained one knockout allele of alpha-synuclein and one flanked by 2 LoxP sites, transgenic cassette NSE-Cre-ER-T2 for regulation by tamoxifen of alpha-synuclein recombination, functional *Sncb* (beta-synuclein), and non-functional *Sncg* (gamma-synuclein).

Mouse experiments were maintained under standard specific pathogen-free (SPF) vivarium conditions, which consisted of a 12 h light/dark cycle, a room temperature of 20–24 °C, and a relative humidity of 30–65% with food and water ad libitum. The procedures were conducted in accordance with the guidelines set forth in Directive 2010/63/EU on the protection of animals used for scientific purposes and approved by the local ethics review committee (83 from 11 September 2024) and ARRIVE guidelines with recommended efforts to minimize the number of the animals and their suffering.

### 2.2. Genotyping

For genotype verification DNA from animals’ ear biopsies (30 mg) was analyzed by PCR using specific primers for gene modifications (Table 1) by the standard amplification program with HS polymerase and 30 cycles: 95 °C (15 s), 60 °C (20 s), and 72 °C (30 s) (Mastercycler nexus 22331, Eppendorf AG, Hamburg, Germany).

For the *Snca* gene, the presence of a 406 b.p. product indicated the flox-site allele, while the 280 b.p. product—knockout allele and 354 b.p.—the wild-type allele in the mouse genome. For the *Sncb* gene, products were detected as 300 b.p. for the knockout allele and 320 b.p. for the unmodified part. The amplification products for the *Sncg* gene were 397 b.p. for the knockout allele and 490 b.p. for the wild-type allele.

For NSE-Cre-ER-T2-cassette determination in the hemizygous and homozygous genotype, real-time quantitative PCR (RT-PCR) was used as described previously [56].

### 2.3. Experimental Design

**Figure 2:**
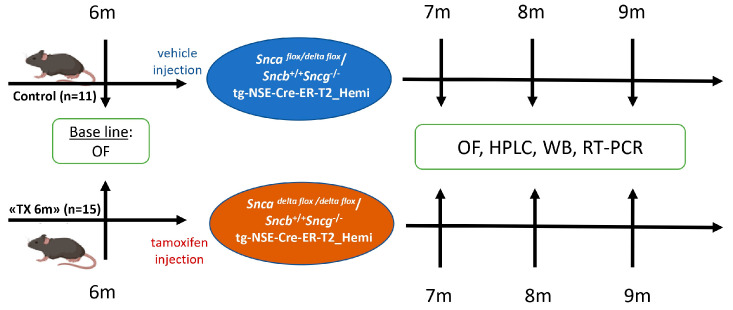
A study flowchart. Induction of *Snca* inactivation by tamoxifen injection at 6 months age and open field (OF) animal testing at 7, 8, and 9 months, after which some of the animals were euthanized. The samples were then analyzed by high-performance liquid chromatography (HPLC), Western blotting (WB), and real-time PCR (RT-PCR).

Experimental cohorts were composed from male mice; the total number of animals was 26, including 11 animals in the control group and 15 in the “TX6m” group. At the age of 6 months, the animals of the “TX6m” group were i.p. injected with tamoxifen–corn oil solution (T5648, Sigma-Aldrich, St. Louis, MO, USA) in a 100 mg/kg dose over 5 consequent days (details in Figure 2). At defined time-points—1, 2, and 3 months after *Snca* inactivation—the animals were euthanized by cervical dislocation, and the bilateral dorsal striatum and midbrains were dissected on dry ice and stored at −80 °C.

### 2.4. Open Field Test

For the assessment of exploratory activity after a 30 min adaptation period in the test room, the animal was placed in a light-gray polyvinyl chloride 40 cm × 40 cm box with a matted bottom for 30 min (OpenScience, Krasnogorsk, Russia). The center of the arena was illuminated at the same level as the home cage (20 lux), while the corners were illuminated at 15 lux. Before each session the internal surface of the box was cleaned with 70% ethanol to reduce the pheromone background. The results were analyzed using EthoVision XT 11.5 software (Noldus, Wageningen, The Netherlands). The average speed throughout the test, frequency of exits to the arena center, and time of animal activity were calculated.

### 2.5. Protein Level Assay

The levels of proteins were analyzed in the samples of the dorsal striatum and midbrains by Western blotting as described early [54,55]. The samples were resolved by 14% PAA gel for alpha- and beta-synucleins and SNAP25 and 16% for analyzing synaptophysin and syntaxin-1a levels. For detection of the antibody–antigen complex, Pierce ECL Western blotting substrate (Thermo Scientific, Rockford, IL, USA) and X-ray film (CL-XPosure™, Thermo Scientific, Rockford, IL, USA) were used. Bands visualization and counting was performed using Vision Works LS software, version 8.0 (UVP, London, UK).

#### Primary and Secondary Antibodies

Mouse monoclonal antibodies against alpha-synuclein, clone 4D6 (ab1903, Abcam Limited, Waltham, MA, USA) at a 1:1000 dilution, rabbit monoclonal antibodies against beta-synuclein (ab76111, Abcam Limited, Waltham, MA, USA) at a 1:2500 dilution, mouse monoclonal antibodies against tyrosine hydroxylase, clone TH-2 (T1299, Sigma-Aldrich, St. Louis, MO, USA), in a 1:1000 dilution, mouse monoclonal antibodies against synaptophysin, clone 2 (BD 611880, BD Biosciences; Transduction Laboratories, San Jose, CA, USA) in a 1:5000 dilution, rabbit monoclonal antibodies against syntaxin-1a (PA11042, Thermo Scientific, Rockford, IL, USA) in a 1:2000 dilution, mouse monoclonal antibodies against SNAP-25, clone 20 (BD 610366, BD Biosciences; Transduction Laboratories) in a 1:5000 dilution, and mouse monoclonal antibodies against beta-actin, clone AC-15 (A5441, Sigma-Aldrich, St. Louis, MO, USA), in a 1:5000 dilution, were used.

Antibodies against mouse immunoglobulin G (IgG) conjugated with horseradish peroxidase (HRP) at a dilution of 1:5000 (517 8-2504, Bio-Rad AbD Serotec Limited, Hercules, CA, USA) and antibodies against rabbit IgG (HRP-conjugated) at a dilution of 1:5000 (519 6-2504, Bio-Rad AbD Serotec Limited, Hercules, CA, USA) were used as secondary antibodies.

### 2.6. Striatal Neurochemical Analysis by High-Pressure Liquid Chromatography (HPLC)

The tissues were homogenized in 0.1 n HClO4 (Sigma Aldrich, St. Louis, MO, USA) with 250 pmol/mL internal standard 3,4-dihydroxybenzylamine (DHBA) hydrobromide (Sigma 240 Aldrich, St. Louis, MO, USA), and then samples were centrifuged for 20 min at 2000× *g* for debris precipitation. HPLC separation was carried out using a reversed-phase column ReproSil-Pur, ODS-3, 4 × 100 mm with a pore diameter of 3 μm (Dr. Majsch, Entringen, Germany) at +30 °C and a mobile phase speed of 1 mL/min supported by a liquid chromatograph LC-20ADsp (Shimadzu, Kyoto, Japan). The electrochemical detector Decade II (Antec Leyden, Zoeterwoude, The Netherlands) equipped with a working glassy carbon electrode (+0.85 V) and a Ag/AgCl reference electrode (the composition of the mobile phase was described previously [53]) was used.

After this, the release time in the standard solution of peaks of interest and the internal standard were identified. For the calculation of the monoamine concentrations, the internal standard method with a calibration curve and LabSolutions 5.87 software (Shimadzu, Kyoto, Japan) was used. The concentration of DA was calculated as the ratio of the area of the DA peak in the sample to that in standard solution (*a*) multiplied by the ratio of the area of the DHBA peak in standard solution to that in the sample (*b*) in standard solution with 250 pmol/mL standard concentration (*M*) in a 0.400 mL volume of striatum sample (*V*):

$$\frac{a}{b}\;\times \;\frac{c}{d}\;\times \;M\;st.sol.\;\times \;V\;str,$$

The concentration of DA metabolites was calculated in a similar way.

Striatal samples were normalized to total protein. The levels of total protein in the samples were analyzed by Pierce (Pierce™ BCA Protein Assay Kits, Thermo Scientific™, Rockford, IL, USA) according to the manufacturer’s instructions. For the analysis of results, the Cytation 3 imaging reader (BioTek, Winooski, VT, USA) and Gen 5.3 program were used.

### 2.7. Analysis of Gene Expression

The prefrontal cortex samples were snap-frozen in dry ice and kept at 80 °C. Total RNA was isolated from tissues with an Extract RNA (Evrogen, Moscow, Russia) according to the manufacturer’s instructions. After homogenization the RNA was precipitated with chloroform in a 1:5 ratio. All the following manipulations were made at 4 °C; all centrifugation was carried out at 12,000 g/rcf. After centrifugation for 15 min, the upper aqueous phase was mixed with 1:1 volume of 100% isopropanol. The tubes were shaken, and the samples were incubated for 10 min at room temperature and centrifuged again for 10 min. The supernatant was removed, the pellets were washed with 75% ethanol by centrifugation for 5 min at maximal speed. This step was repeated twice. Finally, ethanol was removed from the tubes, and the pellet was air-dried.

The concentration of RNA was determined with a NanoDrop 2000 spectrophotometer (NanoDrop Technologies, Wilmington, DE, USA), and 500 ng of total RNA was reversely transcribed using the First Strand cDNA Synthesis MMLV Kit (Evrogen, Moscow, Russia) according to the manufacturer’s instructions.

For the RT-PCR amplification reaction, the buffer of SYBR Green I intercalate dye and Hot Start polymerase 5X qPCR mix-HS SYBR (Evrogen, Moscow, Russia) was used. Each sample was analyzed in triplicate using a 96-well Biorad CFX96 real-time PCR instrument (Biorad, Hercules, CA, USA). All samples were run in triplicate, and data was normalized to GAPDH mRNA expression and calculated as relative-fold changes compared to the control group. The primer sequences used, and amplicon sizes are presented in Table 2.

### 2.8. Statistics

Statistical analysis of data was performed using GraphPad Prism 10.4.3. (San Diego, CA, USA). All data are presented as means ± SEM. Statistical processing of the data was carried out using one-way ANOVA or nonparametric Kruskal–Wallis with post hoc Sidak’s multiple comparisons test and two-way ANOVA with Geisser–Greenhouse correction.

## 3. Results and Discussion

**Figure 3:**
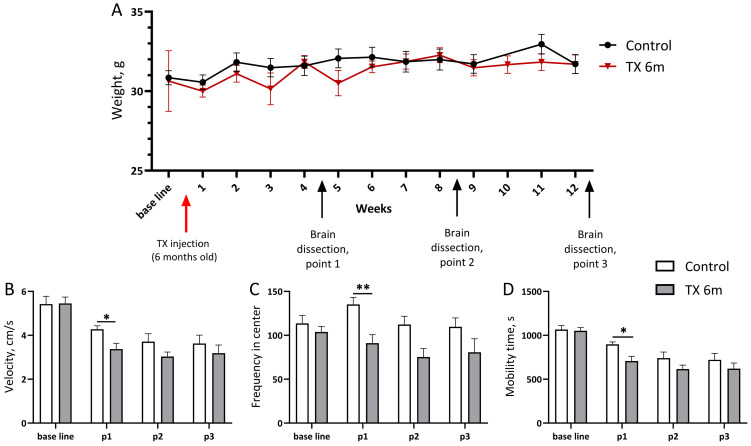
Monitoring of animals’ weight and exploratory activity throughout the experiment. Weight assessment (**A**) was carried out once before tamoxifen (TX) injections and then every week after injections over 6 months. The average movement speed (**B**), frequency of exits to the arena center (**C**), and animal mobility time during the test (**D**) were analyzed in a 30 min open field test at 1, 2, and 3 months after *Snca* gene inactivation. The number of animals analyzed: base line and point 1 (p1)—15 for TX and 11 for control group; p2—12 and 11; p3—9 and 11, respectively. Bar charts show mean ± SEM. * *p* < 0.05, ** *p* < 0.01, two-way ANOVA with Geisser–Greenhouse correction (**A**) and Kruskal–Wallis with post hoc Sidak’s multiple comparisons test (**B**–**D**).

Conditional knockout of the alpha-synuclein coding gene in mice lacking gamma-synuclein was achieved at 6 months old by tamoxifen injections in some of the male mice with the *Snca* flox/delta flox/*Sncb*^+^/*Sncg*^−^/tg-NSE-Cre-ER-T2_Hemi genotype, while the others received oil vehicle (control group). During all experiments, animals’ weight was monitored once a week. No significant changes were found after *Snca* inactivation (Figure 3A).

**Figure 4:**
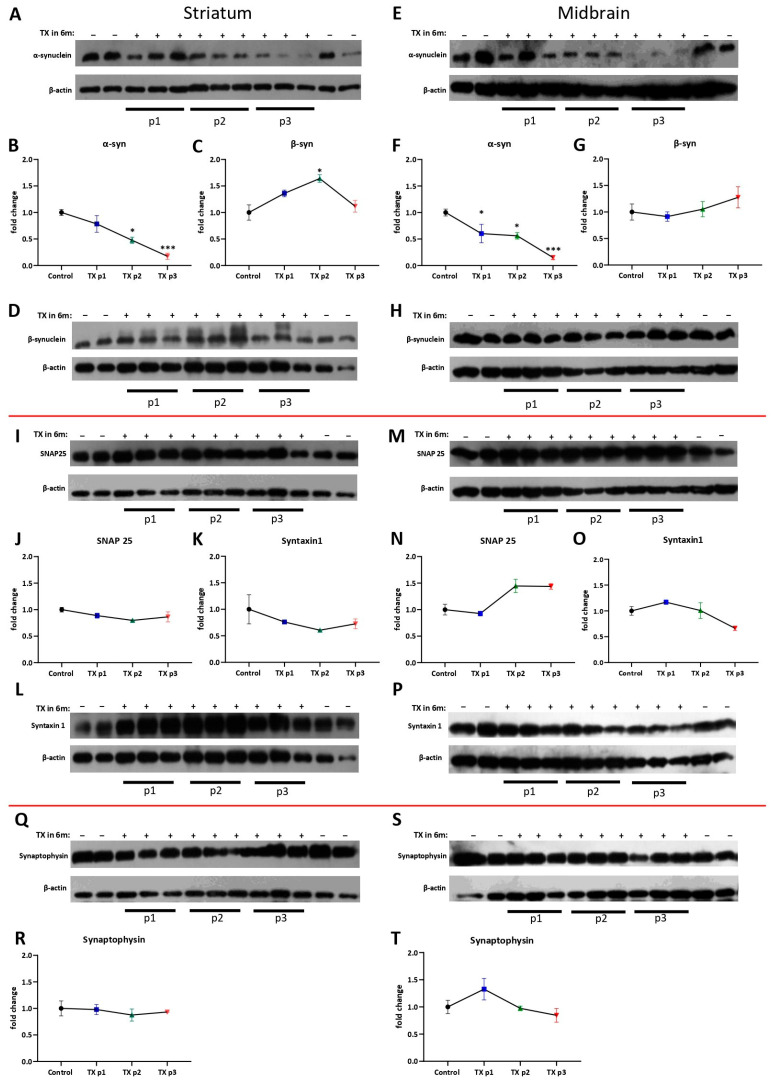
Analysis of presynaptic protein content in mice lacking alpha- and gamma-synucleins. Protein levels were assessed in the dorsal striatum (**A**–**D**,**I**–**L**,**Q**,**R**) and midbrain (**E**–**H**,**M**–**P**,**S**,**T**) at 1, 2, and 3 months after conditional inactivation of *Snca* by semiquantitative immunoblotting. Proteins (molecular weight): Alpha-synuclein, 16 kDa; Beta-synuclein, 18 kDa; SNAP-25, 25 kDa; synaptophysin, 33 kDa; syntaxin, 33 kDa; β-actin, 43 kDa. For quantification, protein levels were normalized to the corresponding β-actin levels (bottom panel) and are presented as fold change relative to the control group. The reference protein b-actin (bottom panel) for D and L, I and Q, and H and M is duplicated because of Figure 4’s design. The number of animals: 3 at each time-point in the TX group and 4 for the control. Bar charts show mean ± SEM. * *p* < 0.05, *** *p* < 0.001, Kruskal–Wallis with post hoc Sidak’s multiple comparisons test.

**Figure 5:**
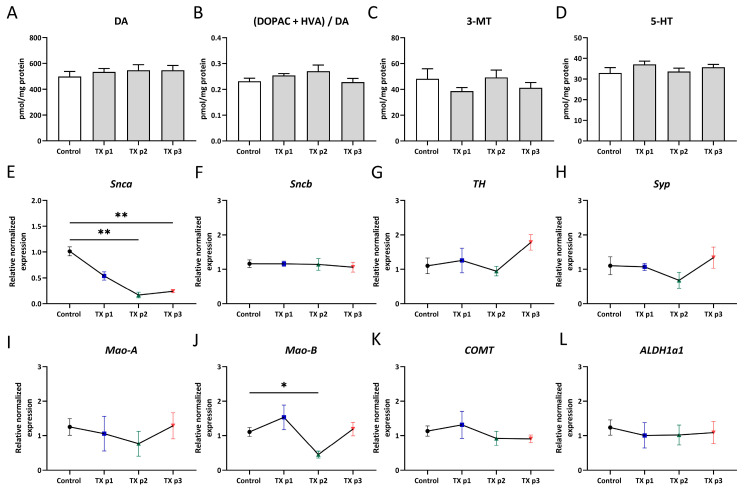
Dopamine metabolism (dorsal striatum) and gene expression of dopamine-related enzyme (midbrain) were evaluated at 1, 2, and 3 months following conditional *Snca* inactivation. (**A**) Dopamine level, (**B**) DOPAC and HVA ratio to dopamine, (**C**) 3-methoxytyramine (3-MT), (**D**) 5-hydroxytryptamine (5-HT) or serotonin level, (**E**–**L**) gene expression levels of alpha- and beta-synuclein, TH, Mao-A, Mao-B, COMT, ALDH1a1, and specific neuron marker synaptophysin. The number of animals per group was TX (p1: *n* = 3, p2: *n* = 3, p3: *n* = 9) and control (*n* = 11). Data are presented as mean ± SEM. * *p* < 0.05, ** *p* < 0.01, Kruskal–Wallis with post hoc Sidak’s multiple comparisons test.

Every month until the age of 9 months, several animals from each group were sacrificed, and the dorsal striatum with the midbrain was dissected for analysis. Alpha-synuclein residual level was assessed by Western blotting with an antibody specific to mouse alpha-synuclein. Compared to the animals of the control group in which alpha-synuclein level was high during all check points, the “TX 6m” group showed a gradual decrease in alpha-synuclein protein in both analyzed structures (Figure 4A,B,E,F). As expected, the level of alpha-synuclein in the striatum became almost undetectable (*p* = 0.0104 at the second point and *p* = 0.0006 at the third, Kruskal–Wallis) at the age of 9 months. Gene expression of *Snca* measured in midbrains was also decreased in the “TX 6m” group at 9 months old (*p* = 0.005, Kruskal–Wallis) (Figure 5E).

Since synuclein proteins play a crucial role in dopamine transmission, alterations to which may cause cognitive and motor symptoms, firstly we evaluated how the lack of two synucleins affects behavioral phenotype. Mice lacking gamma-synuclein were analyzed in a 30 min open field test at 6 months old as the zero point and, later, by longitudinal testing at 1, 2, and 3 months after *Snca* inactivation. At the first point of analysis, the animals of the “TX 6m” group demonstrated a decrease (*p* = 0.0324, two-way ANOVA) in velocity, a reduced frequency of the center arena entries (*p* = 0.0090, two-way ANOVA), and less moving time (*p* = 0.0220, two-way ANOVA) compared to the control group (Figure 3B–D, respectively). The absence of significant exploration deficits in the “TX 6m” group at the next test points show that reduced activity has a transient character, occurring at the early stage after *Snca* inactivation. Data obtained on 10-month-old animals also showed no differences in activity compared to the control group; spatial memory in this age in the Morris water maze test stayed intact as well. No significant differences were observed in the Morris water maze performance of single gamma KO mice [57]. Together these data underline the pivotal role of beta-synuclein in spatial memory formation mechanisms. However, single gamma KO mice exhibited a reduction in peak activity following amphetamine administration compared to wild-type animals [58], which may be due to both decreased dopamine stores and its reduced presynaptic release in the absence of gamma-synuclein.

Surprisingly, no corresponding alterations in dopamine content (Figure 5A) measured by HPLC in the striatum were observed through all check points, although, an increasing trend in the metabolite-to-dopamine ratio had appeared by 8 months in age (Figure 5B). This ratio (DOPAC+HVA/DA) indicates dopaminergic activity and shows how quickly dopamine is being broken down into its metabolites. Thus, in our case it can reflect a trend toward a more rapid breakdown of dopamine. There were no significant differences in striatal DOPAC, HVA level, or (DOPAC/DA) or (HVA/DA) ratios (Supplementary Figure S1). 3-MT is involved in striatal intracellular signaling pathways and its levels regulated by Mao-B and COMT activity, showed a slightly similar trend by gene expression (Figure 5C,J,K). Downregulation of alpha-synuclein also does not enhance serotonin neurotransmission (Figure 5D). We presume that this is the result of the sample size in the group, which was too low for the HPLC method. In that case, since the dopamine level has stayed constant, the increased metabolite-to-dopamine ratio is a consequence of rising dopamine metabolite levels. To confirm this, the expression of *Syp*, *Mao-A*, *Mao-B*, *COMT*, and *ALDH1a1* genes were measured in the midbrain, where DA neuron bodies are concentrated (Figure 5G–L). The expression of *TH* (Figure 5G), the rate-limiting enzyme of dopamine biosynthesis, was the same as in the control group, which was in line with the unchanged dopamine level.

Among dopamine catabolism enzymes, no changes in expression were detected, and only Mao-B levels (Figure 5J) were decreased exactly at 8 months in age (F = 3.038, *p* = 0.0103, Brown–Forsythe and Welch ANOVA tests). Although Mao-B is expressed primarily in astrocytes and not directly within dopaminergic cells [59], it balances the DA concentration of the synaptic cleft in the striatum. As a result, a change in its level can apparently affect DA catabolism. Mao-B inhibitors are used to alleviate PD symptoms, although for strong symptomatic patients, the therapy is effective only in concert with L-DOPA, suggesting a possible role of Mao-B activity in the pre-symptomatic stages of PD [60,61]. In a cell study by Kensuke Kakiuchi et al. showed that Mao-B inhibitor selegiline elevated secretion of endogenous alpha-synuclein compared with basal secretion without intracellular level changes [62]. In accordance with this data, the decrease in Mao-B in our study can be a physiological reaction aimed at restoring alpha-synuclein levels. However, the reduced mouse activity in our study seems to contradict the behavioral disinhibition in Mao-B KO mice [63].

One of the most frequent non-motor symptoms of PD is apathy, occasionally accompanying depression [64]. Nevertheless, the contribution of the serotoninergic system to this kind of symptom in PD has attracted little attention so far. To confirm whether serotonin is involved in behavioral changes in our study, its level was also measured (Figure 5C). However, during all experiments there was not any fluctuation in its concentration.

Alpha-synuclein as a chaperon of the SNARE complex is needed for dopamine release in the synaptic cleft [65]; thereby, it could be possible that in the condition of progressive alpha-synuclein depletion, an assembly will be altered. We analyzed the levels of the vSNARE synaptophysin and tSNARE syntaxin-1 and SNAP-25 proteins as the main components of the complex in the striatum and midbrain by Western blotting, expecting a decrease in levels of some of these proteins in the “TX 6m” group. However, their levels remained constant at all ages in both structures (Figure 4I–T). Could it be a consequence of substitution properties by beta-synuclein in the absence of other family members? To answer this question, the level of beta-synuclein in the striatum and in the midbrain was determined using Western blotting (Figure 4C,D,G,H). Indeed, a significant increase (Figure 4C,D) in beta-synuclein level (*p* = 0.0276, Kruskal–Wallis) was observed at the second point in dorsal striatum samples of the “TX 6m” group compared to the control. Levels of beta-synuclein in the midbrain measured by Western blotting were not changed (Figure 4G,H), which correlated with the *Sncb* expression level determined by RT-PCR (Figure 5F). It is possible that an increase in beta-synuclein level appears firstly as a reaction to sudden alpha-synuclein depletion. Gradually, due to beta-synuclein functional replacement, adaptation to low alpha-synuclein level occurs, and, as a result, the beta-synuclein level returns to the basic stage. This process may characterize synapse reconstruction in the early stage of alpha-synuclein dysfunction with gamma-synuclein absence.

In summary, our novel synuclein KO mouse model offers a perspective for the future characterization of synuclein protein functions and its compensatory potential against the background of a fully formed population of DA neurons during embryogenesis. Additional investigations into this model are essential to a comprehensive understanding of the data we have presented, including HPLC verification. Generally, the confirmation of functional interactions between alpha- and beta-synucleins forms the basis for a novel therapeutic strategy for the synucleinopathy group, harnessing its protective potential.

## 4. Conclusions

Our data suggest that sudden alpha-synuclein depletion against a gamma-synuclein knockout background leads to an increase in beta-synuclein levels. This upregulation likely represents functional replacement and confirms the existing hypothesis about the substitution of alpha-synuclein functions by beta-synuclein. In vivo results supports his compensatory role: beta-synuclein is able to initiate the formation of a complex between aromatic L-aminodecarboxylase, TH, and VMAT-2, which can have an allosteric effect on DAT. In addition, the presence of beta-synuclein increased the activity of TH [22]. Together with the data obtained in our study, such as the unchanged expression of TH and SNARE complex proteins in mice with alpha- and gamma-synuclein KO, it suggests the existence of mechanisms through which beta-synuclein is able to maintain dopamine turnover at a functional level in the absence of other family members. Consequently, dopamine metabolism remains intact. However, the initial period preceding this adaptation is characterized by a decrease in explorer activity and decreased expression levels of the *Mao-B* gene. Expression changes can be caused by specific regulation of Mao-B by alpha-synuclein or can be a physiological reaction aimed at restoring alpha-synuclein levels. In general, these observations characterize synapse reconstruction in the early stages of alpha-synuclein dysfunction with gamma-synuclein absence and lays the groundwork for a novel replacement therapeutic strategy in PD.

## Figures and Tables

**Table 1:** Sequences of primers used for genotyping and amplicon size.

Gene Name	Primer Name	Primer Sequence
*Snca*	A_Int1For A_Int1Rev Cre_rev	5′-TGC TGG GCA CAG TGT TGA TTG-3′ 5′-AAA GGC TGG GCT TCA AGC AG-3′ 5′-CAT GAG TAC TTG TGG CTC AC-3′
*Sncb*	bsynUP bsynWT bsynKO	5′AGGACACCACTGGCCCCGAGTCC-3′ 5′-GACGCACGTCCGCACGTCCACCC-3′ 5′-TGCCCCTGAAATGCTGCGCC-3′
*Sncg*	SAUP GDN NeoB	5′-AGT CCT GGC ACC TCT AAG CA-3′ 5′-GGG CTG ATG TGT GGC TAT CT-3′ 5′-GAA GAA CGA GAT CAG CAG CC-3′

**Table 2:** Sequences of primers used for RT-PCR and amplicon size.

Gene Name	Gene	Direction	Primer Sequence	Product Length
*GAPDH*	glyceraldehyde 3-phosphate dehydrogenase	F R	CAC TGA GCA TCT CCC TCA CA GTG GGT GCA GCG AAC TTT AT	111
α-Synuclein	Alpha-synuclein	F R	CTG CCC TTG CCT CTT TCA TTG TGA ACA CAT CCA TGG CTA AAG A	116
*β*-*Synuclein*	Beta-synuclein	F R	CAA GGA AGG CGT CCT CTA TGT ATG CCT GCT CCT TGG TTT TCT	89
*TH*	tyrosine hydroxylase	F R	GCC TCC TCA CCT ATG CAC TC CCC AGA GAT GCA AGT CCA AT	122
*Mao A*	monoamine oxidase-A	F R	TCA CAG GCC ACA TGT TCG AC AAC TCT ATC CCG GGC TTC CA	119
*Mao B*	monoamine oxidase-B	F R	CCA CAT TGA CCA GAC AGG GG TCT TCA TGC CCA AAG CAG GT	107
*COMT*	catechol-o-methyltransferase	F R	ATC CCA GGA CCT TAT CCC CC GTG TCT GGA AGG TAG CGG TC	99
*ALDH*, *A1*	aldehyde dehydrogenase	F R	GGC CTT CAC TGG ATC AAC AC GGG TGA CTC TCT TCA GAT TG	77

## Data Availability

Data are available upon reasonable request. To access data, Dr. Kirill Chaprov (chaprov@ipac.ac.ru) should be contacted.

## Supplementary Materials

The following supporting information can be downloaded at: https://www.mdpi.com/article/10.3390/biomedicines13122866/s1, Figure S1. Dopamine metabolism at 1, 2, and 3 months after conditional inactivation of *Snca*.

## Abbreviations

The following abbreviations are used in this manuscript:

PD	Parkinson disease
DA	dopamine
DA neurons	dopaminergic neurons
SN	substantia nigra
PFC	prefrontal cortex
VTA	ventral tegmental area
TH	tyrosine hydroxylase
L-DOPA	L-dihydroxyphenylalanine
AADC	aromatic amino acid decarboxylase
DAT	dopamine transporter
VMAT-2	vesicular monoamine transporter 2
Mao-A	monoamine oxidase-A
Mao-B	monoamine oxidase-B
ALDH	aldehyde dehydrogenase
GAPDH	glyceraldehyde 3-phosphate dehydrogenase
COMT	catechol-o-methyltransferase
HVA	homovanilic acid
DHBA	3,4-dihydroxybenzylamine
DOPAL	3,4-dihydroxyphenylacetaldehyde
DOPAC	3,4-dihydroxyphenylacetic acid
3-MT	3-methoxytyramine
5-HT	5-hydroxytryptamine or serotonin
SNARE	soluble NSF attachment receptor
VAMP	vesicle-associated membrane protein
SNAP-25	synaptosomal-associated protein, 25-kD
tSNAREs	target plasma membrane SNARE proteins
vSNAREs	vesicle membrane SNARE proteins
CSPα	cysteine string protein
NSE	neuro-specific enolase
ER-T2	estrogen receptor T2
OF	open field test
HPLC	high-performance liquid chromatography
WB	Western blotting
RT-PCR	real-time PCR
KO	knockout
ANOVA	analysis of variance
