# Supplementary material for: Synaptic Changes in Mice Lacking Alpha- and Gamma-Synucleins
Source: Biomedicines. 2025 Nov 25;13(12):2866. doi: 10.3390/biomedicines13122866 (PMC12730289; doi:10.3390/biomedicines13122866)
Supplement: Supplementary file 1 [file biomedicines-13-02866-s001.zip › biomedicines-3957741-supplementary.pdf]

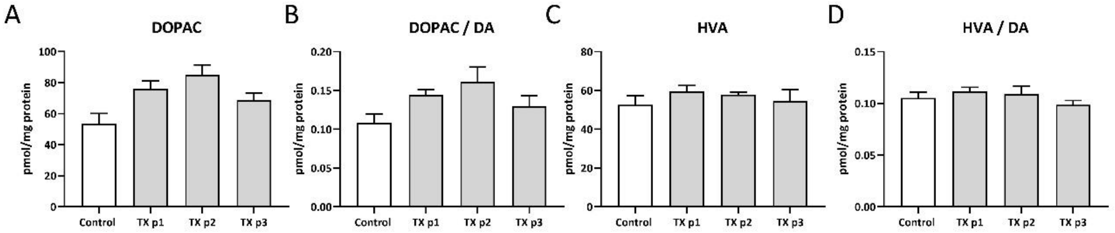

Figure S1. Dopamine metabolism at 1, 2, and 3 months after conditional inactivation of Snca. A – 3,4-dihydroxyphenylacetic acid (DOPAC), B – DOPAC ratio to dopamine, C – homovanilic acid (HVA), D – HVA ratio to dopamine. Graphs show mean ± SEM. Statistical processing of the data was carried out using Kruskal–Wallis with post-hoc Sidak's multiple comparisons test. *p < 0.05, **p < 0.01. No significant differences in striatal DOPAC and HVA level. This ratio (DOPAC/DA) and (HVA/DA) indicates dopaminergic activity and shows how quickly dopamine is being broken down into its metabolites. A higher ratio reflects more rapid breakdown of dopamine, while a lower ratio indicates slower turnover.
